# Editorial: RNA and RNA modification in the pathogenesis, diagnosis and treatment of cancers, Volume II

**DOI:** 10.3389/fonc.2023.1164399

**Published:** 2023-03-10

**Authors:** Qisi Lu, Lihui Wang, Jian-ye Zhang, Dong-Hua Yang

**Affiliations:** ^1^ Department of Hematology, Foresea Life Insurance Guangzhou General Hospital, Guangzhou, China; ^2^ Department of Pharmacology, Shenyang Pharmaceutical University, Shenyang, China; ^3^ Guangzhou Municipal and Guangdong Provincial Key Laboratory of Molecular Target & Clinical Pharmacology, Guangzhou Medical University, Guangzhou, China; ^4^ Clinical Medicine, New York College of Traditional Chinese Medicine, Mineola, NY, United States

**Keywords:** RNA, cancer, pathogenesis, diagnosis, RNA modification

This Research Topic “*RNA and RNA modification in the pathogenesis, diagnosis, and treatment of cancers volume II*” focuses on the areas of RNA in cancer development, prognosis, and molecular signaling pathway. We selected seven manuscripts consisting of two review papers and five research papers aimed at introducing the latest findings in both basic research and clinical application in these research areas.

N6-methyladenosine (m6A) modification has been found in almost all cellular transcripts as the most abundant internal modification of RNA (1). The review by Liu et al. indicated the recent studies on m6A regulated by the m6A “writers” (m6A methyltransferases), m6A “erasers” (m6A demethylases), and m6A “readers” (m6A-binding proteins). m6A modification could affect the stability of RNA base pairing and control RNA splicing, exportation, and translation. “Writers” are composed of methyltransferase complexes, such as methyltransferase-like 3 (METTL3), methyltransferase-like 14 (METTL14), and METTL16, a newly discovered m6A writer and reader. As “erasers,” FTO, ALKBH5, and ALKBH3 can selectively remove methylation of m6A in RNA, while YTH, HNRNPs, eIF3, and IGF2BPs can specifically decode m6A-modified targeted RNA for the m6A group to trigger biological functions as “readers.” Since m6A has a key role in chromatin remodeling involved in almost all RNA metabolism, the alterations in “writers, erasers, and readers” eventually participate in different cellular biological processes including signaling pathway transduction, cell malignancy biology, hematopoietic function, and immunity, thus the dysregulation of m6A associated with cancer occurrence, metastasis, tumor self-renewal capacity, cancer metabolic reprogramming, drug resistance generation, and tumor immune microenvironment.

Gastric cancer is one of the most common malignant tumors with a high mortality worldwide (2). As shown above, *METTL3* is an important m6A methylation modification gene facilitating tumor progression which promotes tumor proliferation, migration, angiogenesis, and other pathways. Peng et al. revealed the role of *METTL3* in GC cells by employing m6A microarray and quantitative proteomics to explore its potential effect and mechanism. This study showed that *METTL3* induced significant alterations in the protein and m6A modification profile in gastric cancer (GC) cells, and downregulated proteins, which were enriched in intracellular mitochondrial oxidative phosphorylation (OXPHOS), were significantly associated with oxidative phosphorylation in METTL3 overexpressing GC cells. Highly expressed and highly methylated molecules regulated by METTL3 exhibited a worse prognosis in GC patients with three (*AVEN*, *DAZAP2*, *DNAJB1*) genes.

In another research on RNA modification in gastric cancer (Jing et al.), the authors focus on the role of RNA modification in the tumor microenvironment and its mechanism of how different RNA modifications directly affect the tumor microenvironment. The study reveals RNA-modified signatures that may have a potential role in the TME and in predicting clinicopathological features by identifying three distinct RNA modification clusters and their role in different biological pathways and starkly correlate with the clinicopathological characteristics, immune cell infiltration, and prognosis of GC patients. An RM score system used to quantify and predict the prognostic value of RNA modification in GC was developed by conducting the principal component analysis, and it shows that patients with a high RM_Score were characterized by a higher tumor mutational burden, mutation frequency, and microsatellite instability which were more susceptible to immunotherapy and had a favorable prognosis.

Non-coding RNA (ncRNA), including microRNA (miRNA), long non-coding RNA (lncRNA), circular RNA (circRNA), and ribosomal RNA (rRNA), which can regulate gene transcription and translation and their dysregulation, has been associated with cancer development and progression (3). Rhabdomyosarcoma (RMS) is a soft tissue sarcoma of skeletal muscle differentiation that predominantly occurs in children and adolescents (4). ncRNA plays a specific role in muscle growth and differentiation, and even small amounts of dysregulation would determine muscle differentiation. Ramadan et al. introduced a brief and general overview of the different classes of ncRNA implicated in RMS and discussed how the dysregulation of ncRNA associated with incomplete myogenic differentiation characterizes RMS cells as well as their enhanced proliferation and metastatic propensity. Extensive research indicates a potential application of ncRNA among disease diagnosis, prognosis, and therapeutic targets in RMS.

Another lncRNA study by Ye et al. investigated the relationship between lncRNA and gefitinib metabolism in non-small cell lung cancer (NSCLC). Metabolized in the liver by cytochrome P450, gefitinib is effective in the treatment of locally advanced or metastatic EGFR-mutated NSCLC patients (5). In this research, the authors screen out gefitinib metabolism-related lncRNAs and explore their prognostic effects, immune microenvironment, and drug sensitivity in NSCLC. Univariate, least absolute shrinkage and selection operator (LASSO), and multivariate regression screening significant genes were used to construct prognostic models. The TME and drug susceptibility were investigated based on risk score data. Differentially expressed lncRNAs were selected for GO/KEGG analysis. The IMvigor210 cohort was used to validate the prognostic model. Finally, the differences in stemness indexes were analyzed. Overall, 13 gefitinib metabolism-related lncRNAs were identified for the construction of prognostic models for NSCLC patients.

Chronic hepatitis B virus (HBV) infection remains the leading cause of hepatocellular carcinoma (HCC), which is another common malignancy with poor outcomes worldwide. RNA-binding proteins (RBPs) are evolutionarily conserved proteins that can bind their RNA targets through their functional RNA-binding domains which regulate mRNA metabolic processes including pre-mRNA splicing, capping, polyadenylation, RNA modification, transportation, localization, translation, and degradation as well as transcription control by binding to chromatin (6). Xu et al. aimed to construct a prognostic model based on the RBP-related mRNAs for HBV-related HCC patients through the application of Kaplan–Meier survival, univariate, LASSO, and multivariate Cox regression analyses, and five RBP-related mRNAs were found out. Among them, F11, FBP1, and SLC6A13 were downregulated in HBV-related HCC which acted as the protective factors for the prognosis of HBV-related HCC patients, while NXPH4 and PSRC1 were downregulated in HBV-related HCC that served as prognostic risk factors.

The maintenance of efficient translation and stability of mRNA is very important in the development of solid tumors, and it is also of great significance in hematological tumors. N-acetyltransferase 10 (NAT10) is an important regulator of mRNA acetylation involved in the regulation of telomerase activity, DNA damage repair, apoptosis resistance, and cell cycle regulation (7). Zhang et al. found that a high expression of NAT10 was associated with poor prognosis in MM patients. BCL-XL (BCL2L1) was screened out as a significant downstream target of NAT10, and the increase of NAT can improve the stability of BCL-XL mRNA and promote protein translation to suppress cell apoptosis *via* activating the PI3K–AKT pathway.

In conclusion, this Research Topic “*RNA and RNA modification in the pathogenesis, diagnosis, and treatment of cancers volume II*” highlights multiple studies for developing RNA and novel therapeutics for cancer diagnosis and treatment.

## Author contributions

QL wrote the first draft. LW, JZ, and D-HY revised the manuscript. All authors contributed to the article and approved the submitted version.
